# Is There a Role for Sound in Plants?

**DOI:** 10.3390/plants11182391

**Published:** 2022-09-14

**Authors:** Filippo Del Stabile, Vittoria Marsili, Luca Forti, Laura Arru

**Affiliations:** Department of Life Sciences, University of Modena and Reggio Emilia, 42122 Reggio Emilia, Italy

**Keywords:** sound vibration, plant acoustics, sound perception, plant communication

## Abstract

Plants have long been considered passive, static, and unchanging organisms, but this view is finally changing. More and more knowledge is showing that plants are aware of their surroundings, and they respond to a surprising variety of stimuli by modifying their growth and development. Plants extensively communicate with the world around them, above and below ground. Although communication through mycorrhizal networks and Volatile Organic Compounds has been known for a long time, acoustic perception and communication are somehow a final frontier of research. Perhaps surprisingly, plants not only respond to sound, they actually seem to emit sound as well. Roots emit audible clicks during growth, and sounds are emitted from xylem vessels, although the nature of these acoustic emissions still needs to be clarified. Even more interesting, there is the possibility that these sounds carry information with ecological implications, such as alerting insects of the hydration state of a possible host plant, and technological implications as well. Monitoring sound emissions could possibly allow careful monitoring of the hydration state of crops, which could mean significantly less water used during irrigation. This review summarizes the current knowledge on sound perception communication in plants and illustrates possible implications and technological applications.

## 1. Introduction

Plants have been around for a long time, far longer than *Homo sapiens*. *Homo sapiens* arose approximately 300,000 years ago [1], a minimal length of time compared with the age of Earth. The earliest land plants, on the other hand, first appeared in the fossil record millions of years prior to this. Non-vascular plants such as the true mosses, the Bryopsida, for example, first appeared in the Mississippian 340 million years ago and appeared well established by the Permian. However, the earliest bryophytes in the fossil record already have the basic thallus organization possessed by current forms, suggesting the possibility that the bryophytes evolved even earlier than the fossil record suggests [2]. Baragwanathia, which is a relatively complex vascular plant, was confirmed to be of Late Silurian origin, which was approximately 420 million years ago [2]. Fossils of the first flowering plants date back to around 130 million years ago, a milestone not only for their role in the evolution of plants but also for the relationship of plants with insects [3]. The appearance, enormous diversification, and ecological radiation of the angiosperms began during the Cretaceous, between 135 to 65 million years ago, and it represented a very significant alteration to the history of life on Earth. It had vast repercussions on the distribution of other groups of land plants and a great effect on the evolution of ecosystems and species. Today, there are more than 350,000 species of extant angiosperms, which is more than all the other groups of land plants combined [4]. They occupy and dominate an astounding range of habitats, and -as autotrophs- the angiosperms are the base upon which most ecosystems are built [4].

Plants are sessile and photoautotrophic, which means that they produce new biomass from CO_2_ using light energy in a process called photosynthesis. Most of the energy that enters terrestrial habitats is the result of photosynthesis. Plants are so important that they go so far as to influence the atmosphere and climate, and yet at the same time, the environment itself has a profound impact on photosynthesis and plants.

And therein lies the point. Plants are not passive organisms, though their sessile nature might make them appear to be static or unchanging. Though plants have an awe-inspiring impact on most life on earth, most people tend to underappreciate or not notice the plants living around them. Plants are alive, and like all things that are alive, they perceive many environmental and physiological signals, and through these, they perfect and modify their growth and development. Not only that, but recent scientific studies have also shown that plants are capable of exhibiting learning, memory, and even intelligence (although maybe not consciousness) [5,6,7,8]. It is reasonable to assume that plants have evolved to do so. After all, as stated before, land plants first appeared millions of years ago. As new plant forms evolved, so did the capacity to perceive stimuli and adapt to a changing environment [9].

But plants do much more than just perceive and react. Plants communicate amongst themselves and with animals. Plants do so in many ways, both above and below ground; a growing body of research shows that plants can even detect and emit sounds (for a review, see [10,11]).

## 2. Communication through Sound

A growing body of research is showing that plants detect and emit sounds. This is unsurprising considering that there is no habitat colonized by plants that is without sound and taking into account their sessile nature and their age on Earth, it is reasonable to think that they have learned to do so.

### 2.1. What Is “Sound”

Briefly, sound is defined as a series of longitudinal waves of pressure that propagate through compressible media, such as air, liquids, or solids. Sound waves that fall in the range of frequencies between 20 Hz and 20 kHz belong to the audible sound, which is what a human ear can hear; frequencies below 20 Hz or higher than 20 kHz are defined as infrasound or ultrasound, respectively [12]. However, sound is not only vibration energy; it is also pressure generated by vibration waves that move through a suitable medium in the form of compression and rarefaction [12]. For a sound to be perceived, however, it is not sufficient that it consists of frequencies in the audible range; it must also have sufficient sound pressure, that is, the pressure variation produced by the acoustic phenomenon compared with the static value. For example, in the air a sound pressure of 20 µPa corresponds to a level of sound pressure of 0 dB [13].

Application of sound at different frequencies, pressure levels, duration, and repetition of exposure periods has been proved to have an influence on plant growth, development, and germination [10]. Taken together, these studies belong to the field of Plant Acoustics. Whether or not sound perception and/or emission are used in plant communication is a fascinating field of research. However, for acoustic plant communication to exist, there needs to be an emitter, in other words, a source of sound and a receiver not only able to perceive the signal but also able to decipher and eventually perform a sort of coherent response.

Sound stimulation has been proven to switch on stress-induced genes [14] or enhance genes related to disease resistance [15], but there is also a sort of new age interest in growing plants with sound. Traditional ethnic music can positively affect the productivity and quality of plants. Many studies are heading in that direction: Javanese music has been applied to Chinese broccoli (*Brassica alboglabra*) plants [16], and *Desmodyium girans* (Telegraph plant) [17] and rice (*Oryza sativa*) [18] show better growth performance when exposed to Buddhist pirith chants.

### 2.2. Sound at Cellular and Subcellular Level

On a cellular level, it seems that the most likely candidate for sound signaling is Ca^2+^, which acts as a second messenger to Sound Vibrations (SVs). Although direct evidence is lacking, it is possible that SVs activate plasma membrane channels, evoking a membrane potential-based signaling cascade. Several studies have shown an efflux/influx of Ca^2+^ following SVs. It was shown, for example, that Chrysanthemum cells treated for one hour with SVs of 100 dB and 1000 Hz had an increase in H^+^-ATPase activity [19]. The upstream component of this increased activity was found to be Ca^2+^. It appears that transient increases in cytosolic Ca^2+^ concentrations lead to an activation of calcium-dependent protein kinases, which then activate the H^+^-ATPases. These kinases go on to regulate proteins and transcription factors, therefore altering gene expression [20,21,22,23]. For example, after treatment with SVs, plant cells showed increases in α-amylase activity and as a result, an increase in sugar levels. ROS scavenging enzymes have been shown to increase activity after SV treatment, and overall, the most common plant response to SV treatment is increased growth, for example, through increased cell division. All this, however, is just a general overview of what happens at the cellular level following sound perception, and it is well described in greater detail by Mishra and co-workers in 2016 [23].

But there is another side to the coin. Sound is generated by vibrating objects, and the components of eukaryotic cells do just that. Following the hydrolysis of Adenosine triphosphate (ATP), motor proteins such as myosin generate vibrations [24]. In addition, the nanomechanical motions of the cell wall of *Saccharomyces cerevisiae*, baker’s yeast, are in the range of 800–1600 Hz, with amplitudes of 3 nm. Interestingly, exposing the cells of *S. cerevisiae* to a metabolic inhibitor caused the periodic motion to cease [25]. Cells are surrounded by other cells so that a cell can be influenced by the mechanical properties of its adjacent cells, and this can build up to a collective mode which results in amplification of the signal [24].

### 2.3. Sound like Touch

Sound and touch have similar physical properties, and yet plants cannot only properly distinguish between sound and touch, but they are also able to distinguish between relevant and irrelevant sound. The ability of plants to perceive touch has been well known for a long time. One need only to look at the carnivorous plant *Dionaea muscipula* or at *Mimosa pudica* to see plants reacting to touch. Since sound is generated by a vibrating body, and it propagates longitudinally by vibrating the particles of the medium, it passes through, when it reaches a body, it vibrates the body mechanically as well. In other words, sound waves mechanically impact an object they meet on their path [9].

The molecular basis for the perception of a mechanical stimulus in plants remains to be identified. However, touch sensitivity is not just limited to sensitive plant and carnivorous species. Every plant (or plant cell) perceives and accordingly responds to mechanostimulation [26]. Even plant roots are extremely sensitive to touch, being able to turn around an obstacle in their path. Like wind, light, rain, touch, sound is a pressure wave that translates into a mechanical influence. For the perception of a mechanical stimulus in plants, Telewski suggested a “unified hypothesis of mechanoperception in plants” [27] with two models of mechano-receptors: (a) a cytoskeleton based on plasmodesma—plasma membrane—cellular network and (b) ion channels activated by stretching. Given the similarity of the sound stimulus to that of touch, it has recently been seen that signals and perception mechanisms of these two stimuli are common. However, the plants seem to distinguish between the two well in an extraordinarily sophisticated way [26].

## 3. Effects of Sound Perception

Human conversation typically has an intensity of approximately 60 dB, and at this intensity, it can elicit vibrations, for example, in hearing organs, of just 10–50 nm. At these scales, the mechanical energy imparted by the vibrations is exceedingly small, and yet we have no problems hearing during conversation. Considering this, it really is reasonable to think that something as small as a trichome could vibrate in response to SVs and possibly convey information [28].

In much the same way plants have adapted to different pollinators, plants have adapted to different sounds in their environments. For example, flower morphology affects the efficiency of pollinators, affects the way pollinators visit flowers and the success of pollen import and export [29]. Similarly, the carnivorous pitcher plant *Nepenthes hemsleyana* could possibly have evolved pitchers that reflect the echolocation of bats. The plant *N. hemsleyana* has a mutualistic interaction with bats, supplying a safe, parasite-free roosting spot, and the bats in return fertilize the plant with nitrogen-rich droppings, enhancing the nitrogen uptake of these plants by an average of 34% [30].

### 3.1. Buzz Pollination

Insects, primarily Hymenoptera [31], use vibrations to extract pollen from a wide variety of flower morphologies with poricidal anthers, that is, anthers where the pollen exits the anther through an apical pore or slit. This phenomenon is known as buzz pollination. In poricidal anthers, the pollen is not freely accessible, and its removal requires vibration. As many as 8–10% of angiosperms possess poricidal anthers that are pollinated through the use of vibrations. Interestingly, buzz pollination seems to have arisen independently several times in about 65 plant families [32]. Buzz pollination is not limited to a specific flower morphology, although it seems that the Solanum type flower has evolved specifically in response to sonicating bees. Flowers with poricidal anthers are visited by many insects, even non-sonicating insects that chew through the anthers to reach the pollen, but the primary visitors are sonicating bees [31].

Sonication seems to have arisen in a common ancestor of bees during the early Cretaceous [32]. A bee lands on a flower and curls with the ventral side of the body around the anthers in a C shape, with the wings tightly folded back over the abdomen during sonication [31,33]. The bee then rapidly contracts the thoracic muscles while preventing the wings from beating. The vibrations are transmitted to the anthers, which resonate, transmitting energy to the pollen, which is then expelled through the apical aperture [31]. Centrifugal forces are generated, which eject the pollen [34].

There are both insect-related and plant-related variables that affect buzz pollination. Vibrations produced by sonicating bees can be characterized by duration, amplitude, and frequency. It was found that the greatest effect on pollen removal from anthers was given by duration and amplitude, while frequency had only a weak effect on pollen removal. Moreover, heavier bees produced buzzes with greater amplitude, ejecting more pollen [35]. The magnitude of the vibration required to eject pollen from the anthers increased with frequency. The vibration frequency determines the time that a force may act on a particle, and therefore higher frequencies require higher amplitudes [34].

In terms of duration, bees increased the duration of their buzzing when visiting virgin flowers, and buzzes were shorter when returning to flowers that had already been visited. This could suggest that bees adjust the duration of their buzzing in relation to the pollen content of the flower [31]. In theory, if a bee vibrated for a long enough time, it could extract all of the available pollen [34].

The frequency of the buzzing is under physical and physiological control rather than behavioral control. This is because the vibrations depend on the muscles of the bee, and therefore there are limits to the frequencies they can achieve. The peak frequency, which refers to the frequency with the greatest relative energy within a buzzing vibration, varies between 100–400 Hz depending on the species of bee. Through harmonic frequencies, which are positive integer multiples of the original peak frequency (sound-standing waves), frequencies as high as 2000 Hz can be reached [31], but as stated before, this has very little effect on pollen removal. The optimal peak frequencies do, however, vary among plant species but still remain under 1000 Hz.

Plant traits also affect buzz pollination. Plant structures can either enhance or dampen the amplitude of the vibrations. For example, rigid, multi-layered anthers release more pollen compared with flexible anthers when vibrated. It’s reasonable to think that the size of the apical pore influences the amount of pollen released [31].

### 3.2. Sweetened Nectar

Yet another example in the realm of pollination is the production of sweeter nectar within as little as three minutes following the perception of sound by flowers of *Oenothera drummondii*. The flowers of *O. drummondii* mechanically vibrated in response to recordings of bees and moths flying and also vibrated in response to the flight of a live bee, showing the same increase in nectar sugar content [36]. The volume of nectar remained the same, meaning that an increase in sugar concentration was not a result of a drop in water content. The velocities of the oscillations of the flowers that in this experiment caused an increase in sugar concentration in the nectar was found in other experiments to be able to elicit defense responses by plants [36]. Interestingly, the vibration of the flowers depended on the presence of petals, as flowers that had their petals removed or flowers covered by glass ceased to show a response to the sound vibrations.

### 3.3. Interpreting Relevant and Irrelevant Sounds

These examples show the important ecological role that sound can play in a plant’s life. Plants don’t live isolated from the rest of the world. Instead, there are extensive connections with other plants, animals, and microbes. Around plants, there are rich communities of arthropods, many of which use vibrations to find mates or prey. For example, vibrations caused by the chewing of *Plathypena scabra* worms caused predatory *Podisus maculiventris* stinkbugs to begin their search [37]. Chewing herbivores produce specific high-amplitude vibrations that travel quickly to other parts of the plant, and this can produce a local and systemic response in other parts of the plant. *Arabidopsis thaliana* leaves exposed to recordings of caterpillars chewing were proved to be primed for defense [38]. The plants that had been exposed to chewing vibrations showed higher levels of glucosinolates and anthocyanins following herbivory, while there was no increase in anthocyanins in the plants that either received no vibrations or received vibrations from recordings of leafhopper singing or recordings of the wind [38]. Interestingly, as with the greater amplitude of bee buzzing increasing pollen removal, higher amplitudes induced higher amounts of aliphatic glucosinolates [38]. It is still to be understood how the response caused by the vibrations of a herbivore can generate an induced resistance or a systemic resistance (for an in-depth study, see [38]). One possibility is that the plant subject to herbivory integrates the vibrational signal with others coming from the herbivore’s attack. As plants perceive warning signals via VOCs from nearby stressed plants, and VOCs can serve as a sort of chemical language in the communication between plants [39], also vibrations can be used at least in some cases in plant communication [24,40,41]. This is yet another example of the ecological role that sound can play in a plant’s life. The fact that plants perceive sound from so many different sources and adapt proves them to be ingenuously aware of their environment.

## 4. Sound below Ground

Sound travels easily and far in dense substrates, and soil is a wonderful example. Since the epigeal part of plants does respond to sound, it might be strange that the roots of plants would not be able to do the same, especially considering that vibrations in the soil are present at all times and places. A possible example of the hypogeal part of plants responding to sound vibrations could be the frequency selective behavioral response of *Zea mays* roots. When exposed to a continuous sound, the root tips very clearly bend towards the source of the sound [42]. Furthermore, the root tips very clearly show different responses to different frequencies, with the biggest response elicited by a continuous sound of frequency between 200–300 Hz. Interestingly, the root tips generated acoustic emissions, which could be measured at some distance in the hydroponic medium in which the roots were growing [42].

*Pisum sativum* roots showed a behavioral response to sound. Even in the absence of moisture, the roots of *P. sativum* were able to locate water thanks to the vibrations induced by the movement of the water [6]. Interestingly, in the presence of moisture, the authors showed that the roots preferred the moisture over the acoustic emissions, suggesting that plants could use the sound of water flowing to locate water and then more accurately find the water using moisture gradients. Interestingly, the roots showed avoidance behavior when in the presence of sound equipment, even when the sound equipment was broadcasting the sound of water flowing. The authors hypothesized that the roots were able to sense a cue, such as magnets in the speakers, that directed their growth away from the sound equipment [6].

## 5. Can We Communicate with Plants by Means of Sound

Very clearly, sound has a very important ecological role in the lives of plants, but sound vibration treatment can also be used commercially. For example, treatment of harvested tomatoes with sound was shown to delay ripening. Mature green tomatoes were treated with sound waves of 250, 500, 800, 1000, and 1500 Hz for 6 h. All of the sound treatments except the 800 Hz and the 1.5 kHz delayed tomato ripening, with the 1 kHz treatment having the biggest effect. Seven days after treatment with the 1 kHz sound wave, 85% of the treated tomatoes were still green, whereas over 50% of the non-treated tomatoes had turned red [43].

The treatment with sound waves was shown to decrease both ethylene production in the treated tomatoes and the respiration rate. By the time the respiration rate of the non-treated tomatoes had begun falling after the completion of the ripening process, the respiration rate in the treated tomatoes was still increasing, suggesting that ripening was indeed delayed. Furthermore, the change in color from green to red was more gradual in treated tomatoes compared with non-treated tomatoes. Finally, the flesh firmness of treated tomatoes decreased more gradually, whereas the flesh firmness of the non-treated tomatoes dropped sharply after five days [43]. This last result could possibly be explained with the help of a previous study which found that treatment with sound waves decreased the deformability of plant cell membranes and made them more rigid. The sound waves seemed to have an effect not on the cell membranes themselves but seemed to cause microfilaments to rearrange and become more rigid. Interestingly, different frequencies had different effects on the deformability of the cell membranes, with higher frequencies causing the deformability to decrease [44]. The possibility of delaying ripening through sound wave treatment has important ramifications for lengthening the shelf life of products such as tomatoes.

Treatment with sound can also act as a plant growth stimulant, although the underlying mechanisms for this increase in growth have not yet been properly identified. A possible explanation for this is the fact that sound treatment alters plant growth regulatory hormone levels. Sound treatment increases IAA and decreases ABA levels, and this could be a factor in promoting plant growth [10]. It was found, for example, that following treatment of Chrysanthemum cells with 1000 Hz, 100 dB sound soluble protein content increased significantly compared with a control group. The treatment lasted for 60 min each day, and the treated plants were separated into groups treated for 3, 6, 9, 12, and 15 days. A rich content of soluble proteins is the basis for many physiological activities. Interestingly, soluble protein content increased significantly after six days and even more significantly after nine days but actually dropped back down when the treatment carried on too long, such as the 12- and 15-day treatments. Furthermore, sugar content increased following stimulation compared with the control group. Finally, amylase activity increased following stimulation compared with the control group. The importance of frequency in the response of plants to sound is shown time and time again. While 1000 Hz sound was shown to be beneficial by increasing soluble protein and sugar content and increasing amylase activity, 2000 Hz sound actually proved to be damaging for plant cells [19].

Other examples of sound treatment as a growth stimulant could be the increased yields in sound-treated tomato or the treatment of wheat with sound waves of 92 dB and 5 kHz to increase yield and dry weight. Photosynthesis was shown to increase following sound treatment in rice and strawberries, and photosynthesis-related proteins were highly expressed following 8-h sound treatments at 250 or 500 Hz in *Arabidopsis thaliana* [10]. Sound increased the resistance of strawberries to insects and disease [23]. SV treatment brought about an increase in the length, number, and activity of Actinidia chinensis roots. Highly dormant seeds of Echinacea Angustifolia showed enhanced germination following treatment with sound vibrations of 1000 Hz and 100 dB [23]. SV treatment has possible applications in biotechnology, ultrasound being able to enhance Agrobacterium-mediated transformation of several plant species, or audible SVs showing to increase in vitro growth of many plant species [45,46,47,48,49,50].

Sound treatment was also shown to induce drought tolerance in *Arabidopsis thaliana*, leading to a significant increase in survival rates compared with control plants [51]. At the end of the treatment, plants were sampled to determine changes in transcription. Eighty-nine genes were found to have had their expression altered, 87 of which upregulated, the remaining two downregulated. Of the 87 upregulated genes, 44 are involved in stress-related responses [51].

### The Case of Cavitation

Considering that plants do respond to sound, could plants themselves actually emit sounds? Going even further, if plants do emit sounds, could other plants, or perhaps the same plant, perceive these acoustic emissions and react to them? As mentioned previously, it was indeed found that corn roots grown hydroponically emitted sounds [42]. However, prior to this discovery, it was already believed that in conditions of drought, cavitation in xylem vessels could be a source of acoustic emissions [9]. Cavitation is the mechanical breakage of the continuous water column in a xylem vessel that occurs when the tensile strength of the water column is exceeded. This is accompanied by the build-up of mechanical pressure, which, when released, leads to elastic wave propagation [11]. In other words, there is an abrupt release of tension in the xylem vessel lumen as the liquid water under negative pressure is replaced by water vapor [52].

Previously the measurement of acoustic emissions following cavitation was done through actual contact between sensors and the plant itself [53]. Although still interesting, this method does not take into account whether these emissions could be sensed at a distance. However, plants do emit airborne sounds that can be detected from a distance. Different tomato (*Solanum Lycopersicum*) and tobacco (*Nicotiana tabacum*) plants were placed in an acoustically isolated anechoic box under different treatments and were recorded simultaneously at a distance of 10 cm by two directional microphones in order to eliminate false detections of clicks caused by the electrical equipment. The plants were either cut, placed under drought stress, or were in control conditions. The plants that were under stress or cut emitted significantly more sounds compared with the control plants. For the drought-stressed plants, the mean number of sounds emitted per hour was 35.4 for tomato and 11.0 for tobacco, while for cut plants, the mean number of sounds emitted per hour was 25.2 and 15.2 for tomato and tobacco, respectively. Surprisingly, the control plants not subjected to either drought or cutting emitted less than one sound per hour [53].

Although the precise values differ slightly for tomato and tobacco, the mean peak frequencies of the emitted sounds, in other words, the frequencies with the maximal energy, were between 49 kHz and 58 kHz. These results not only indicate that the emitted sounds are ultrasonic, that is above 20 kHz and not detectable by the human ear, but also confirm that these emissions are detectable at least at a distance of 10 cm. This means that these emissions could be theoretically detected by other organisms, such as insects or other plants [53].

What makes this experiment especially interesting is the use of machine learning, which refers to a system’s ability to improve and extend itself by learning new knowledge rather than being programmed with that knowledge [54], to determine whether it was possible to identify the condition of a plant based on the sounds it emitted. The regularized machine learning classifier, which was also trained to discriminate against the electrical noises made by the recording equipment, was able to correctly identify the condition of the plants based on the sounds they emitted. Not only could it distinguish between the control plants and the treated plants, but it could also distinguish between the cut plants and the drought-stressed plants. This is fascinating because it could mean that the sounds that plants emit when under drought stress could carry information; therefore these sounds could be intercepted by other organisms who could then respond and adapt [53]. Finally, tomato plants were placed in a greenhouse to simulate more realistic conditions [53]. The recording equipment was trained to discriminate between tomato sounds and greenhouse sounds. A consistent acoustic pattern was found in that the number of sounds emitted is very low when the plant has been recently irrigated, but the number of emissions drops as the plant becomes dry.

These ultrasound emissions could be detected at a distance of 3 to 5 m. This means that it is possible that these acoustic emissions could be perceived by other organisms. For example, many moths that use tomato and tobacco plants as hosts for their larvae can perceive sounds in the frequencies and intensities that were detected in this experiment. It is possible that the information contained in the acoustic emissions of drought-stressed plants could inform these moths not to lay their eggs on these plants [53]. The emission of sound by drought-stressed plants has important implications in agriculture, as the detection of these sounds could be used to monitor the water status of crops, and this could, in turn, lead to more efficient and precise irrigation, therefore reducing water usage.

Interestingly, it seems that the acoustic emissions of plants can actually be distinguished between low-dB Ultrasonic Acoustic Emissions (UAE), which are below 27 dB, and high-dB UAE (above 35 dB) associated with cavitation. Most investigations on acoustic emission detection have focused on higher dB ranges under the assumption that low dB sounds cannot be distinguished from background noise. However, it was found that signals in the low dB range seem to have a consistent pattern. UAE remained in the low dB range on sunless days and at night and transitioned abruptly to the high dB range on sunny days. High dB acoustic emissions coincided sharply with decreased sap flow rate in *Quercus pubescens* [52].

Typically, low dB acoustic emissions increase in intensity as bark tissue expands with hydration, so at night or while it is raining, the intensity of the low dB emissions increases. During the day, as transpiration occurs, the diameter of the stem shrinks as water is lost, and the intensity of the low dB emissions decreases gradually until there is a very abrupt transition to the high dB emissions that are probably caused by cavitation. The highest low dB acoustic emissions occur before dawn, which is when the least water movement occurs. So low dB acoustic emissions closely follow stem radius changes de-trended for growth. There are different possible origins for the low dB emissions [52].

One possible source for the low dB sound could be the mechanical noise of the stem shrinking and expanding. Or it could be the respiration and metabolic growth activity of the cambium and ray parenchyma cells. These obviously produce diurnal courses of CO_2_ efflux from the stem. If the water content is high enough, then the respiration rate follows the temperature. At low water contents, however, the missing water seems to inhibit biochemical activity, regardless of temperature. In conditions of drought, respiration follows stem water content more closely and is largely independent of temperature. Under such conditions, low dB acoustic emissions and stem water content match respiration. When the turgor pressure in the cambium increases, for example, at night, radial growth occurs; consequently, respiration increases, and low dB acoustic emissions increase [52].

For a long time, it was thought that sounds generated by plants were always a product of cavitation, but the overabundance of sound emissions by plants makes it highly unlikely that all sounds generated by plants are a product of cavitation, considering the limited number of water-conducting elements. Although it seems clear that cavitation can indeed emit sound, some authors believe that sounds generated from the xylem area are not caused by cavitation but by a stable bubble system capable of transporting water through peristaltic waves. Laschimke and colleagues believe that acoustic emissions are a result of sudden surface rearrangements of groups of wall-adherent microbubbles under positive pressure. These microbubbles, which have also been photographed, are largely stable and do not immediately result in embolism [55]. Laschimke also found, in *Ulmus glabra*, that acoustic emissions are incessant during transpiration and re-hydration. Therefore it is very unlikely that all acoustic emissions are a result of cavitation. Acoustic activity is undiminished during the night. This means that acoustic activity is not solely a result of transpiration, although transpiration does modify the type of activity, as stated before. The authors analyzed the waveforms of the acoustic emissions in *U. glabra* during a testing period of 77 h. By analyzing the waveform, it is possible to better understand the underlying physiological processes that cause the acoustic emission [56]. It is reasonable to expect that a sound emitted by a cavitation event would have a very rapid fading of the acoustic signal, as the water column is rapidly and violently retracted along the vessel following the disruption. However, very few of the 2200 acoustic emission events had a waveform profile of this type. Instead, most acoustic signals showed great variability in the duration, amplitude, and frequency, which can hardly be explained by the cavitation theory of acoustic emission.

## 6. Plant Alerts

### Communicating Drought Stress

At this point, it is clear that not only do plants respond to sound, but also that plants emit a wide variety of acoustic emissions, with varying frequencies, from audible to ultrasound, and varying durations and intensities. However, it is harder to actually pinpoint the source of these emissions, and the theory that acoustic emission was a result of cavitation has been put into question, or at least it has been shown that not all emission is a result of it.

As stated before, sound treatment increased drought tolerance in *A. thaliana*. The same was shown in *Oryza sativa*. Different rice plants were treated with single frequencies of 0.25, 0.5, 0.8, 1.0, and 1.5 kHz for 24 h. After this treatment, the plants were placed under drought stress for five days. Sound treatment with frequencies of 0.8 kHz and above increased stomatal conductance, relative water content, and quantum yield of PS II. Furthermore, hydrogen peroxide production was inferior in these plants, and the temperature of the sound-treated plants and leaves was inferior compared with control [57]. So, could it be possible that the acoustic emissions by plants could be perceived by other plants? It has already been mentioned that certain moths can detect sound in the frequencies emitted by drought-stressed tomato and tobacco plants and possibly avoid laying their eggs on those stressed plants; a machine learning tool could very clearly distinguish between stressed and control plants. Could a drought-stressed plant emitting cavitation sounds, among other sounds, alert other plants of impending drought stress? This is a possibility.

Freeze-thaw cycles are the second most important reason for inducing cavitation, so it was natural for studies to focus on the acoustic emission of plants following such cycles. A study found that ultrasonic acoustic emissions are detected during the freezing part of the cycle in conifers, occurring during the ice formation part of the cycle, and most UAE are perceived during the first freeze-thaw cycle, with lower emissions during subsequent cycles. It was also found that samples with water contents close to dehydration emitted UAE during temperature cycles, whereas very dehydrated samples or saturated samples showed few UAE [58].

But why should plants communicate through the use of sound? What possible advantages could be obtained through the use of sound, as opposed to the use, for example, of VOCs? Firstly, physical signals such as sound can propagate very rapidly, as opposed to VOCs that need to diffuse through the air. Moreover, sounds can be analyzed quickly and can be sensed at very low intensities and over long distances. Sound not only propagates a lot faster than volatiles, but it also has the added benefit of allowing for more accurate source localization. This means that sound has features that degrade predictably over distance, allowing a receiver to estimate the distance from the emitter. Not only that, but acoustic emission is also the result of a physical process, at least in the case of cavitation, which means that there is little to no energy cost involved [24]. VOCs, on the other hand, represents a significant loss of energy, and a substantial amount of the carbon fixed by plants is re-emitted into the atmosphere through VOC communication [59]. A possible advantage of VOCs, however, could be their ability to linger in their environment after emission, whereas acoustic signals very obviously dissipate extremely quickly. However, it’s also important to note that volatile signals depend on diffusion and wind direction and, therefore, also suffer from their dilution. This means that although VOCs can linger, they nevertheless need to be present in sufficient quantities to be able to be perceived. Sound, on the other hand, can be perceived by organisms even at very low intensities [24].

## 7. Conclusions

At this point, it should be clear that plants respond and emit sound in a wide variety of intensities, frequencies, and durations, as a result of different mechanisms. This is unsurprising in a way, considering the age of plants on Earth and the omnipresent distribution of sound. More study on the acoustic emission of plants is needed to properly understand just what causes sound in plants and how many possible sources of sound there are. A better understanding of sound emission could also clarify other mechanisms, such as cavitation following freeze-thaw cycles and, in general, what happens inside a plant during stress in a non-invasive and real-time manner.

A common theme in studies focusing on the role of sound in plants is the scarce knowledge of the molecular and cellular mechanisms of sound perception and signal transduction. More research at this level of the plant system could determine just how plants react and produce sound, and this could help clarify the ecological role of sound communication in plants. After all, specialized receptors or proteins involved solely in sound perception have yet to be identified in plants. Further research can also clarify the differences and similarities, if they exist, between sound and mechanical stimulation. After that, considering the similarities in essence, the propagation, and in the effects between touch and sound, could light mechanical stimulation cause the same reactions as sound treatment?

Studies on sound perception in plants need to continue also because they have important implications in agriculture and biotechnology. Being able to accurately assess the water status of crops through their acoustic emissions can lead to more efficient irrigation, for example. Sound can also be used to increase the shelf life of products, increase yields, or to trigger plant defenses against pathogens. However, if sound treatment of plants begins widespread use, it is also important to consider its potential side effects on animals, humans, and plants. After all, realizing that plants respond to sound means also accepting that noise pollution affects the plant world as well. Sound treatment on plants needs to be wary of increasing noise pollution problems.

Plants have long been considered to be unchanging, passive, and static organisms, but this view needs to change. Plants are far more ingenious and aware than initially thought, and changing the way we view plants can lead to research that can better take into consideration their capabilities.

## Data Availability

Not applicable.
